# Microsatellite Repeat Instability Fuels Evolution of Embryonic Enhancers in Hawaiian *Drosophila*

**DOI:** 10.1371/journal.pone.0101177

**Published:** 2014-06-30

**Authors:** Andrew Brittain, Elizabeth Stroebele, Albert Erives

**Affiliations:** Department of Biology, University of Iowa, Iowa City, Iowa, United States of America; University of Iceland, Iceland

## Abstract

For ∼30 million years, the eggs of Hawaiian *Drosophila* were laid in ever-changing environments caused by high rates of island formation. The associated diversification of the size and developmental rate of the syncytial fly embryo would have altered morphogenic gradients, thus necessitating frequent evolutionary compensation of transcriptional responses. We investigate the consequences these radiations had on transcriptional enhancers patterning the embryo to see whether their pattern of molecular evolution is different from non-Hawaiian species. We identify and functionally assay in transgenic *D. melanogaster* the Neurogenic Ectoderm Enhancers from two different Hawaiian *Drosophila* groups: (*i*) the picture wing group, and (*ii*) the modified mouthparts group. We find that the binding sites in this set of well-characterized enhancers are footprinted by diverse microsatellite repeat (MSR) sequences. We further show that Hawaiian embryonic enhancers in general are enriched in MSR relative to both Hawaiian non-embryonic enhancers and non-Hawaiian embryonic enhancers. We propose embryonic enhancers are sensitive to Activator spacing because they often serve as assembly scaffolds for the aggregation of transcription factor activator complexes. Furthermore, as most indels are produced by microsatellite repeat slippage, enhancers from Hawaiian *Drosophila* lineages, which experience dynamic evolutionary pressures, would become grossly enriched in MSR content.

## Introduction

**Figure 1:**
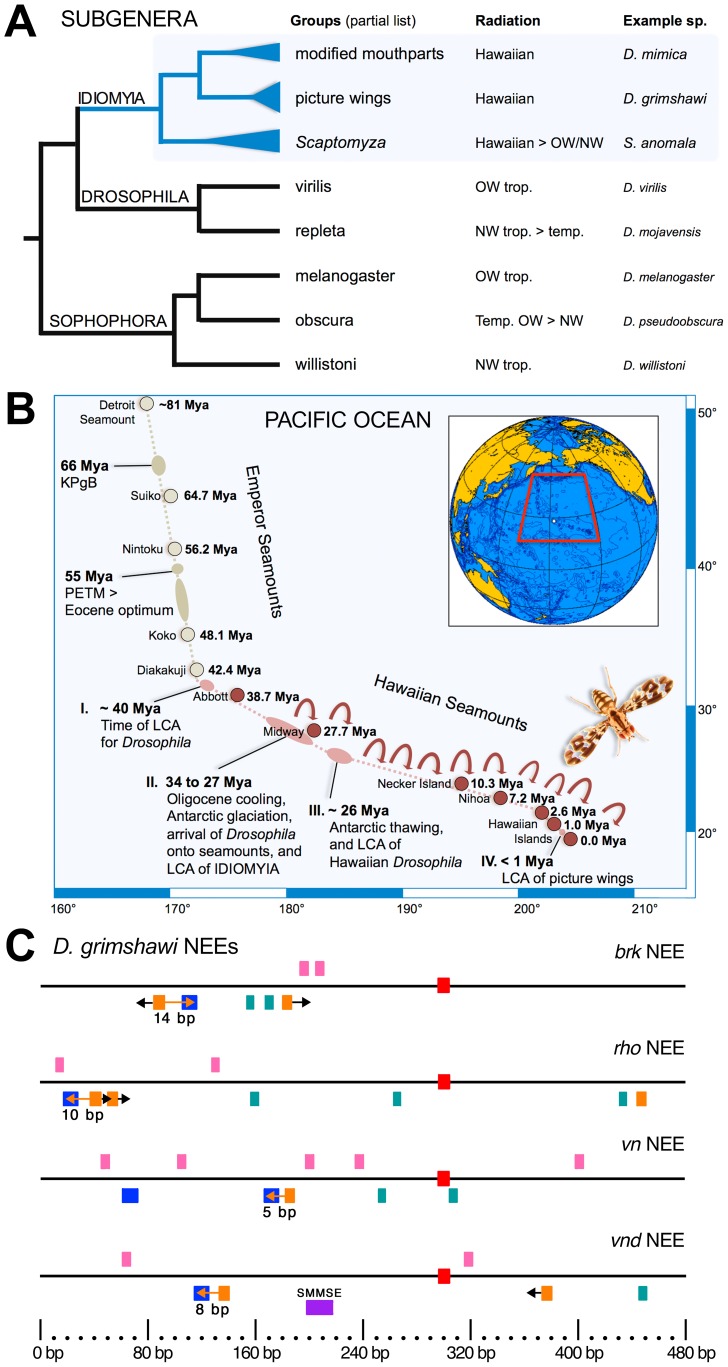
The Neurogenic Ectoderm Enhancers from the Hawaiian species *D. grimshawi*. (**A**) Shown is a phylogenetic tree showing the relationship of the Hawaiian *Drosophila*, which comprise subgenus IDIOMYIA along with the so-called *Scaptomyza* [16]. This subgenus gave rise to multiple clades (highlighted in blue) corresponding to multiple adaptive radiations associated with the last ∼30 My history of island formation. Embryonic enhancers from two of these clades, the modified mouthparts group and the picture wing group, are analyzed in this study. (**B**) Shown is a map of the Hawaiian Seamounts with relevant events in the evolutionary lineage leading to the Hawaiian *Drosophila* groups indicated by the ages of the various islands. The picture wing group (inset shows an example *D. grimshawi* adult fly) is one of the most recent radiations and is closely associated with the newest and easternmost front of the seamounts, *i.e*., the Hawaiian islands. (**C**) The graphs indicate the site architecture of the four genus-canonical Neurogenic Ectoderm Enhancers (NEEs), which we identified in the *D. grimshawi* genome. The NEEs are a class of early embryonic enhancers downstream of the Dorsal morphogen gradient [4], [5], [19]. The search for NEEs in the *D. grimshawi* genome was conducted by searching for linked sites for the Twist:Daughterless bHLH heterodimer (Twi:Da) and the rel homology domain-containing TF Dorsal (5′-CACATGT 0–41 bp GGAAABYCC), plus a nearby Su(H) site binding site located up to +/−300 bp away (see Material & Methods). Three horizontal tracks indicate sequences matching different TF binding motifs (tracks are separated to avoid overlap and for ease of visualization when viewed in black & white print or with color-blindness). Above line: pink boxes = Snail (5′-CARRTG) [57]. On line: red boxes = Su(H) (5′-YGTGRGAA). A single Su(H) site is present in each enhancer and is used to anchor each sequence at basepair position 300 bp [the Su(H) motif matches the top strand for all except in the *vn* NEE]. Below line: orange boxes = Twist:Daughterless (Twi:Da, 5′-CACATGT); blue boxes = Dorsal (5′-VGGAAABYCCV); blue and orange boxes connected by arrow = linked Twi:Da–Dorsal sites with text indicating spacer length; and purple boxes = Shnurri:Mad:Medea Silencer Element (SMMSE). The Schnurri/Mad/Medea Silencer Element (SMMSE), which functions to constrain the dorsal border of activity for the NEE at *vnd* [19], matches the *D. melanogaster*/*D. grimshawi* consensus 5′-MYGGCGWCACACTGTCTGS and is highlighted in purple.

Genomic sequences from twelve ecomorphologically diverse *Drosophila* species have been assembled [1] and studied [1]–[6]. One of these twelve species, *D. grimshawi*, is from the large “picture wing” group, which itself is one of many groups of the remarkably speciose Hawaiian *Drosophila,* corresponding to almost 500 of the ∼1500 described *Drosophila* species and others yet to be adequately described [7]–[16]. The Hawaiian species form a monophyletic group and include recent radiations exemplified by the picture wing group, which diverged from a most recent common ancestor less than one million years ago (∼0.5–0.7 Mya), older radiations such as that exemplified by the “modified mouthparts” group, and older still the so-called *Scaptomyza* flies (Fig. 1A). Thus, the *Drosophila* subgenus, known as IDIOMYIA (Hawaiian *Drosophila*+*Scaptomyza*) illustrates the profound species fecundity of the island forming process that in ∼40 million years produced the Hawaiian seamount island chain, which was colonized by *Drosophila* over ∼30 million years ago (Fig. 1B).

We consider the consequences of the sustained pattern of frequent species radiations on transcriptional enhancers of the syncytial fly embryo within Hawaiian *Drosophila*. In this evolutionary context, the evolving *Drosophila* egg is being laid in new and ever-changing environments. The associated evolutionary diversification of the syncytial fly embryo (*viz*., the shape, size, and developmental rate of the embryo as previously shown [17], [18]) would have continuously altered embryonic morphogen gradients of each lineage, thus necessitating compensatory evolution of the gradient-sensing responses of target enhancers [4]. We therefore ask whether the pattern of molecular evolution at developmental enhancers that interpret embryonic morphogen gradients in Hawaiian *Drosophila* differs from that in non-Hawaiian *Drosophila*.

To address this question, we considered a group of complex transcriptional enhancers that are important to *Drosophila* morphogenesis: the Neurogenic Ectoderm Enhancers (NEEs) [4], [5], [19], [20]. Unlike protein-coding gene families, which are related by common descent (*i.e.*, homology), the NEEs in a single genome are similar only by molecular convergence (parallelism) and so we define them as a mechanistic “family”. Four “canonical” NEEs are present as orthologs across the genus in the unrelated loci *rhomboid* (*rho*), *vein* (*vn*), *brinker* (*brk*), and *ventral nervous system defective* (*vnd*) [4], [5], [19]–[21]. The NEEs are responsive to the morphogenic gradient of Dorsal, which patterns the dorsal/ventral axis of the early embryo. While Dorsal is a homolog of the NFkB-enhanceosome forming factor, and is known to work with many different co-activator transcription factors (TFs) along the D/V axis [22], we found that the NEEs contain binding sites for a specific subset of these factors. Binding sites for the activators, Dorsal, Twist, Su(H), and the mesodermal repressor Snail are present in each of the NEEs we have found [4], [20], which is consistent with the NEEs representing one specific equivalence class of enhancers; there exist other lateral stripe enhancers and sometimes even lateral stripe “shadow enhancers” [4], [23]–[26] at the same NEE-bearing loci, but they feature binding sites for distinctly different sets of factors other than Dorsal.

Here, we identify and analyze the evolutionary divergence of NEEs in one representative species of the Hawaiian picture wing group, which has a fully sequenced genome (*D. grimshawi*) [1], and in one representative species of the Hawaiian modified mouthparts group (*D. mimica*), for which we cloned, sequenced, and tested their enhancers. We show that relative to *D. virilis*, which is a representative of the continental Old World group of the subgenus DROSOPHILA that gave rise to the Hawaiian *Drosophila*, the intervening DNA sequences between the NEE binding sites have been largely replaced by ***microsatellite repeat*** (**MSR**) sequences. This unique MSR-footprint demarcates the functional binding sites for Dorsal, Twist/Snail, and Su(H). It also demarcates the dedicated Snail binding sites and sites for the general embryonic timing factor Zelda. We also demonstrate that relative enrichment of MSR in Hawaiian *Drosophila* is specific to developmental enhancers of embryogenesis and suggests that diverse enhancers function as enhanceosome scaffolds with sensitive spacing requirements. Because Dorsal, Twist, Su(H), and Zelda are all polyglutamine-rich transcriptional activators, we propose a specific model in which enhancers functioning as scaffolds for polyglutamine-mediated co-factor complexes are both sensitive to *cis*-element spacing and are sites of MSR-enrichment when subjected to evolutionary pressures.

## Results

### Neurogenic Ectoderm Enhancers (NEEs) from Hawaiian *Drosophila*

To identify the repertoire of NEE functions in a Hawaiian *Drosophila* species, we used the assembled genome from the Hawaiian picture wing fly, *D. grimshawi* (Fig. 1A, and adult pictured in Fig. 1B inset). Specifically, we searched for a Su(H)-binding motif (5′-YGTGRGAA) located within 300 bp of linked Twist and Dorsal binding sites (5′-CACATGT 0–40 bp nGGAAABYCCn, where the Dorsal site could be in any orientation and the n’s are included here only to indicate the normal extent of the Dorsal binding site; see methods). We find only the four genus-canonical NEEs at *brk*, *rho*, *vn*, and *vnd*, each having linked Dorsal and Twist binding sites with spacers of length 14 bp, 10 bp, 5 bp, and 8 bp, respectively (Fig. 1C). Previously, we showed the length of the spacer separating these linked Dorsal and Twist sites to be a major determinant of the extent to which the NEE is responsive to the Dorsal morphogen [4], [5].

**Figure 2:**
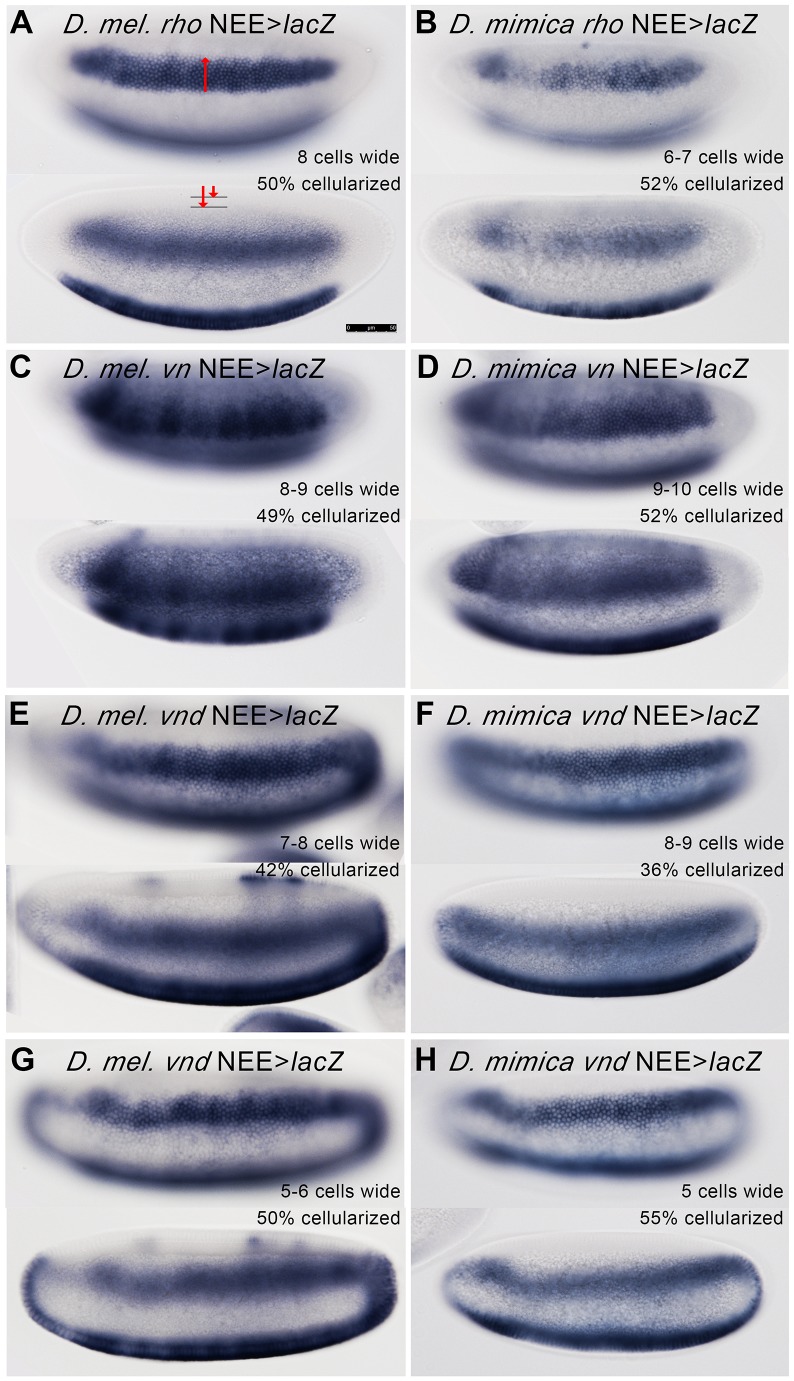
The NEEs from *D. mimica* Can Drive Expression in Neurogenic Ectoderm of *D. melanogaster* Despite Replacement of Inter-Element Spacers with MSR. NEEs from the *D. mimica*, a member of the Hawaiian modified mouthparts clade, were cloned and tested in *D. melanogaster* transgenic reporter assays. Expression of the *lacZ* reporter gene driven from *D. melanogaster* NEEs (**A, C, E, G**) and *D. mimica* NEEs (**B, D, F, H**) as determined by *in situ* hybridization with an anti-*lacZ* probe are shown. In each panel two optical cross-sections are shown. The top image is a surface view allowing determination of the stripe of expression (numbers of cells spanning D/V axis). The bottom image is a cross-section through the dorsal midline allowing determination of the exact stage of embryogenesis (% cellularization as determined at 50% egg length on the dorsal midline). The expression patterns driven by the *rho* (**A, B**) and *vn* (**C, D**) NEEs are shown for the stages close to 50% cellularization. The expression patterns for the *vnd* NEEs are shown at two time points: an earlier time point at about ∼40% cellularization (**E, F**) and a later time point at ∼50% cellularization (**G, H**). For both Hawaiian and non-Hawaiian *vnd* NEEs, activity is dorsally repressed by the the Dpp gradient via a conserved binding site for the Shnurri:Mad:Medea complex beginning at about midway through cellularization [19]. All embryos are oriented with anterior pole to the left and dorsal side on top. The 50 micron scale bar shown in (**A**) is the same for all figures.

We then designed primers on the basis of the NEE sequences in *D. grimshawi*, and successfully amplified intact fragments, ∼500 to 600 bp in length, containing the NEEs from the *rho*, *vn*, and *vnd* loci from a Hawaiian modified mouthparts fly, *D. mimica*, which we have begun culturing in the lab (see Methods). We made standard fusion reporter genes using the −42 *eve*:*lacZ β-tub* 3′-UTR reporter construct in a P-element vector and transformed them into *D. melanogaster* to test for function. All three of these enhancers are *bona fide* NEEs by definition of their site composition and organization, and have discernible neuroectodermal enhancer activity in *D. melanogaster* embryos despite ∼40 million years of evolution in addition to the accelerated levels of evolution in the Hawaiian system (Fig. 2). We find that the *vnd* NEE still encodes a predicted low threshold response that drives early expression, when the Dorsal nuclear gradient is still increasing ventrally [27], but a later dorsally-repressed expression pattern due in part to a well-conserved Schnurri/Mad/Medea silence element [19] (Fig. 2, E–H).

### Extensive Microsatellite Repeat (MSR) Replacement of Hawaiian NEE intersite spacers

**Figure 3:**
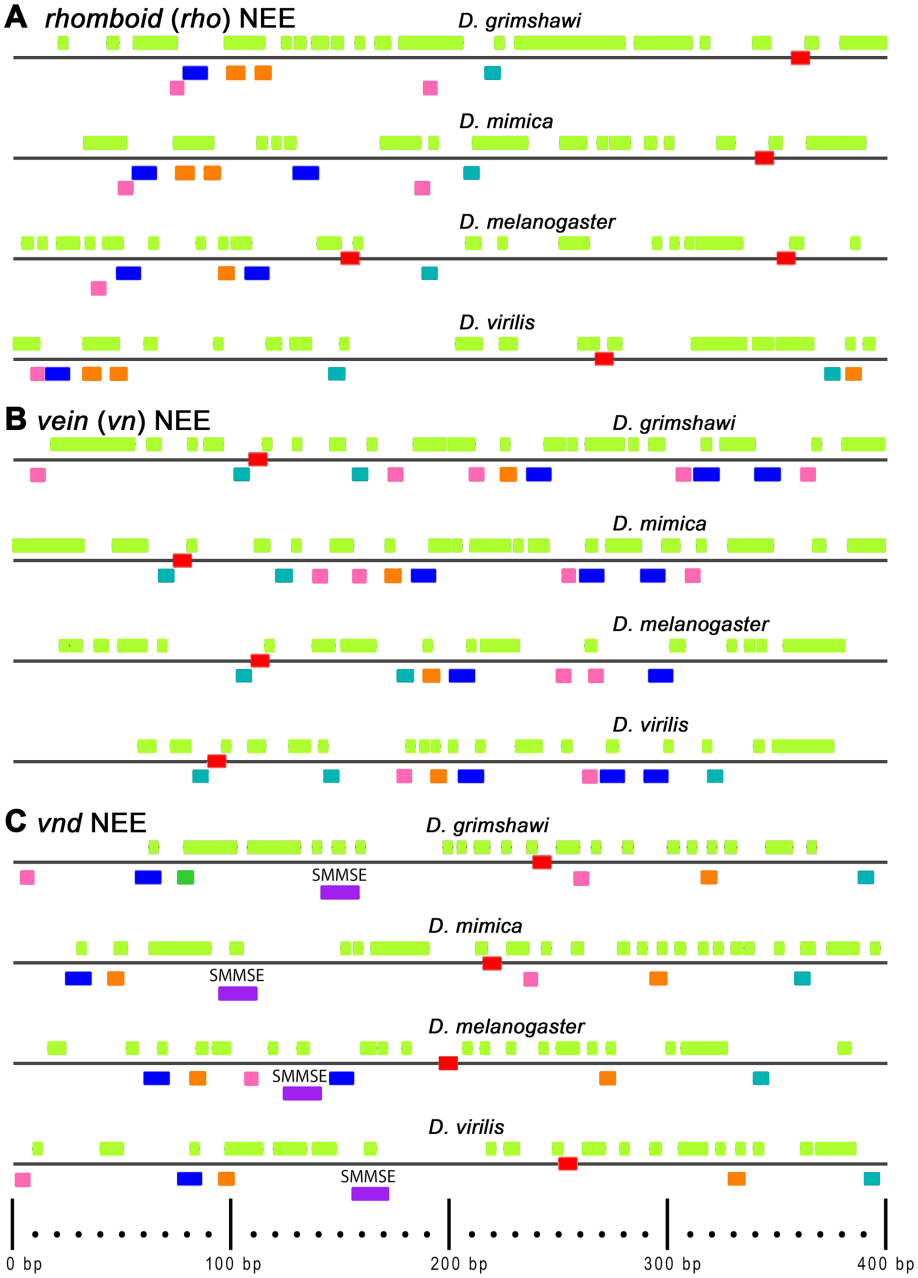
Diverse Direct MSRs Fill the Inter-Element Spacers of NEEs from Hawaiian *Drosophila*. Graph depicts micro-satelleite repeat (MSR) content, also known as simple sequence repeats, for the NEEs of the Hawaiian species, *D. grimshawi* and *D. mimica*, and two non-Hawaiian species, *D. virilis* and *D. melanogaster*, which also represent extremes in genome size (large and small, respectively). Note that *D. melanogaster* is the outgroup species as it is a member of the SOPHOPHORA subgenus. The panels are ordered by orthologous enhancers (*rho*, *vn*, *vnd*) and then by species within each panel, top to bottom. The colored boxes correspond to the same TF binding motifs depicted in Fig. 1 except that the Dorsal *Dβ* motif is relaxed at one position to 5′-VGGAAABNCCV (underlined “N”) in order to match the site in *D. mimica*’s NEE at *vnd*. The MSR content is plotted by a UNIX-type regular expression, “(.{2,50})\1” corresponding to two or more direct repeats of a unit sequence that is at least 2 bp or more in length (green yellow highlight above each line). Many such MSR sequences overlap. While difficult to see at first glance the Hawaiian NEEs are much enriched in this type of content. Exactly 400 bp centered on the NEE heterotypic site cluster is shown.

Inspection of the Hawaiian NEEs reveals diverse microsatellite repeat patterns besides the known genus-wide enrichment of CA-dinucleotide repeats in NEEs [5]. To visualize precisely this content and to determine the possibility of longer repeats being present, we plotted all direct (tail to head) repeats (two or more) of a unit sequence that is 2–50 bp long (fluorescent green boxes in Fig. 3) (also see Materials & Methods). We find that the *rho* and *vn* NEEs from both Hawaiian species are qualitatively enriched in MSR content relative to both *D. melanogaster* and *D. virilis* (Fig. 3A, B). The enrichment that can be seen in comparison to both non-Hawaiians is made more significant by the fact that *D. virilis* has a much larger genome than *D. melanogaster* while also being more closely related to the Hawaiian lineages [4], [5]. The enrichment is not seen in the Hawaiian *vnd* NEEs relative to the non-Hawaiians (Fig. 3C), but this is as expected for the following two reasons. First, the *vnd* NEEs are less variable in activity phylogenetically relative to all other NEEs [4]. Second, the *vnd* NEEs possess a Shnurri:Mad:Medea Silencer Element, which corresponds to a second repressive input from the Dpp morphogen gradient and which ensures its characteristic ventral pattern of expression, critical to its role in patterning the nervous system [19].

**Figure 4:**
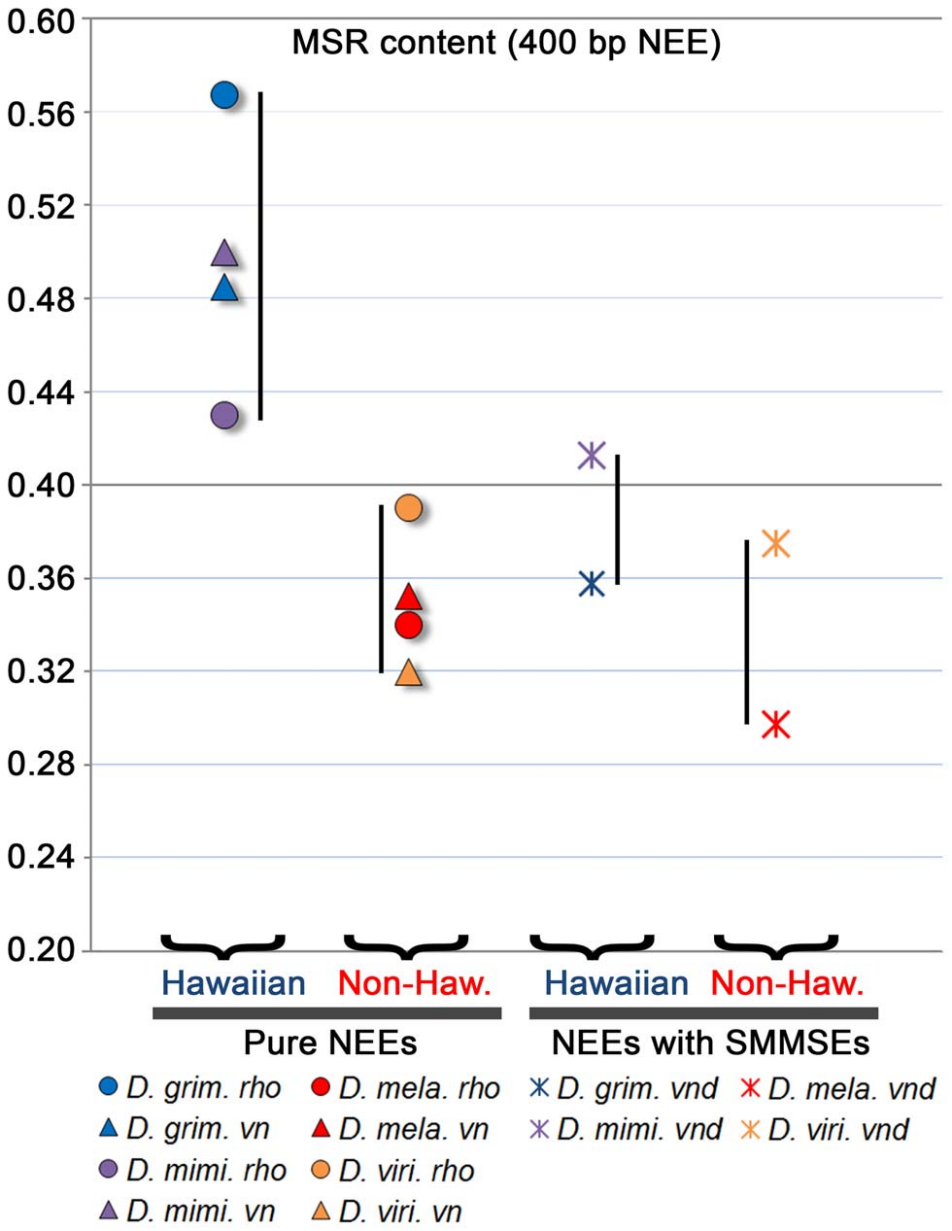
Hawaiian NEEs without SMM silencer elements are enriched in MSR content relative to Non-Hawaiians. Graph plots the MSR content in the 400Figure 3. The points for the “pure” NEEs, which lack Shnurri/Mad/Medea Silence Elements (SMMSE) are plotted separately from the *vnd* NEE, which contains a highly conserved SMMSE [19]. NEEs for Hawaiians and non-Hawaiians are plotted separately for ease of comparison. The *vnd* NEE activity is the least variable in output both ontogenetically and phylogenetically [4]. Its highly constrained early ventral expression is fundamentally important for correct D/V patterning of the nervous system and is thus not likely to be subject to shifting selection for a change in stripe width as we previously demonstrated [19]. Assuming that MSR is a signature of previous selection for changes in threshold responses, these results support the view that MSR content is uniquely enriched in enhancers subject to dynamic evolutionary pressures. Note that the length of the SMMSE that is not also MSR-like is 14 bp (19 bp *minus* 5 bp because of the underlined sequence in the SMMSE motif: 5′-MYGGCGWCACACTGTCTGS.) Thus, the presence of an SMMSE can only account for a reduction of MSR content of no more than 3.5%.

To better quantify the MSR enrichment, we also plotted the exact MSR content for all three of these enhancers across all four species (Fig. 4). This shows that the NEEs without Shnurri/Mad/Medea Silencer Elements (SMMSE [19]) from the Hawaiian species have MSR content in the range of 43–57% in a 400 bp window encompassing all of the relevant TF binding sites (labeled “pure NEEs” in Fig. 4). In comparison, the pure NEEs from the non-Hawaiians have much less content in the range of 32–38%, similar to the range for the *vnd* NEEs of all species.

The striking nature of these enhancers can be summarized as follows. The MSR motifs in the *rho* and *vn* NEEs of Hawaiians (yellow green tracks in Fig. 3) encompass much of the enhancer, and the remaining sequences are either known TF binding sites (for Dorsal, Twist, Su(H), and Snail), or Zelda sites, which are present generally in early embryonic enhancers but have not been specifically pinpointed in the NEEs [28], [29]. Zelda is considered a pioneer TF for early embryonic activation of genes, whose expression is patterned along the D/V and A/P axes [29]–[31].

**Figure 5:**
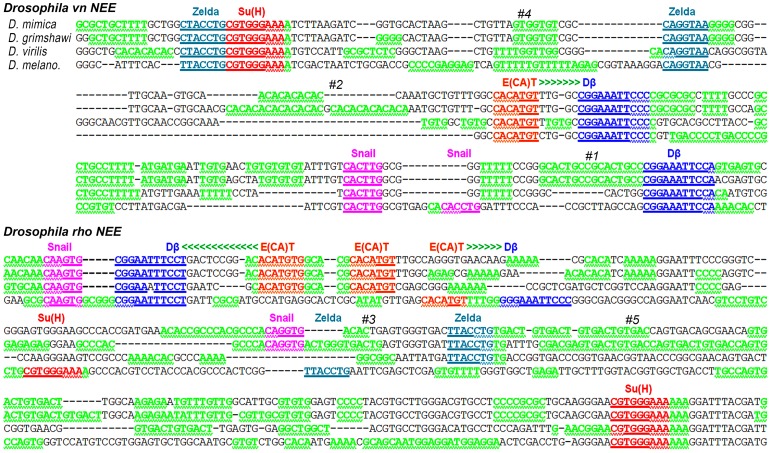
The non-Dpp constrained NEEs at *rho* and *vein* are rapidly diverging via MSR mutagenesis in the Hawaiian lineages. Shown is a sequence alignment for the *vn* (top) and *rho* (bottom) NEEs highlighted to show MSR content (green letters with squiggly underline). The motif coloring follows previous figures. Due to multiple insertions and deletions, these homologous sequences depicted are of different lengths. Numbers indicate the presence of novel MSR sequences present only in Hawaiians (the octapeptide 2x repeat at #1), present only in Hawaiians but still diverging (5 to 13 repeats of CA-microsatellite at #2), sites of MSR repeats of different types at the same position and present only in the DROSOPHILA subgenus (#3) or across the entire genus (#4), and repeats of made of smaller repeats and seen only in Hawaiian species (#5). In addition, other divergent repeat content that is not highlighted by the strict MSR algorithm can also be seen (see Material & Methods).

We list a few examples of Hawaiian MSR enrichment that illustrate the range of repeat patterns and their phylogenetic distributions. There are many examples of both large and small duplicated blocks conserved only in the Hawaiians (Fig. 5, *#1*), and some of these have since diverged in repeat number (Fig. 5, *#2*). In some locations, repeats are found in only one of the Hawaiian species, such as an octamer repeat in the *D. grimshawi rho* NEE, which is a fragment of the Snail/Zelda sites (Fig. 5, *#3*). In other locations, repeats are conserved across the genus but the repeat unit sequence differs indicating a potential region of frequently amplified MSR sourced from different sequences prone to repeat slippage (Fig. 5, *#4*). Last, there are long repeat sequences present in the Hawaiians that are composed of smaller unit repeats (*i.e.*, repeats of repeats; Fig. 5, *#5*). In many places, the repeats are evidently diverging based on changes to the repeat unit or appearance of indels that disrupt their repeat pattern. Thus, molecular drive based on MSR slippage is an important mechanism in NEE evolution at the *rho* and *vn* loci of Hawaiian lineages but its subdued presence in the constrained *vnd* NEEs suggests that natural selection continuously acts on this mutagenic source of functional variation [32].

### MSR-Enrichment in Embryonic *vs.* Non-embryonic Notch-Target Enhancers in *D. grimshawi*

The extreme MSR-footprint is widespread in the Hawaiian NEEs and here we demonstrate that in general the embryonic enhancers from Hawaiian *Drosophila* are enriched for MSR using two different genome-wide analyses described below. We first asked whether this MSR-enrichment was a general property of Notch-target, Su(H) binding site containing enhancers or only a specific feature of embryonic enhancers targeted by Notch/Su(H). To do this, we undertook an analysis of the entire set of Su(H) binding repertoire for *D. virilis* and *D. grimshawi*. We first identified all individual Su(H) sites (5′-YGTGRGAA) and/or clusters of sites in each of the two genomes. This dataset was composed of blocks containing up to 270 bp of sequence flanking the Su(H) site or site cluster when possible, but ∼3% of sequences had less because of close proximity to the edge of a contig but were not eliminated (385/13,473 and 476/14,904 for *D. grimshawi* and *D. virilis*, respectively). Site clusters were defined as having at least two Su(H)-binding sequences separated by <540 bp (*i.e*., less than twice the desired flanking distance of 270 bp). Site clusters defined 6.0% of the data for *D. grimshawi* (815/13,473), and 7.1% of the data for *D. virilis* (1,060/14,904).

In order to identify conserved blocks between Hawaiian and non-Hawaiian Su(H)-binding motif containing repertoires, we identified sequence alignment parameters that are suited for the patterns of indel/MSR-mediated divergence that we see in *Drosophila* enhancers. We took a heuristic approach to settle on a set of customized regulatory “*rblastn*” parameters that gave the highest alignment scores to NEEs that are homologous to each other across different *Drosophila* species (see Methods). Using this *rblastn* pipeline and an E-value cutoff of <1.0e-15, we identified ∼3400 homologous sequences between *D. grimshawi* and *D. virilis*, which includes the canonical NEEs present across the genus at four unrelated loci: *rho*, *vn*, *brk*, and *vnd* [4], [5], [19]–[21].

**Figure 6:**
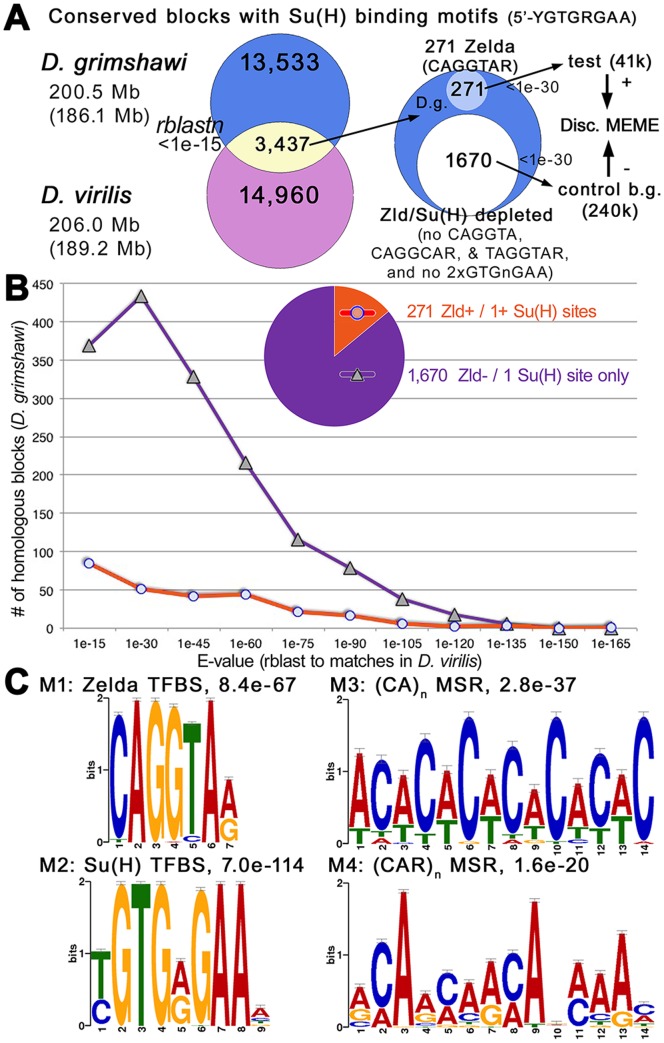
Microsatellite Repeats are Enriched in Conserved Su(H) Site-Containing *D. grimshawi* DNAs of Embryonic Enhancers Relative to Non-Embryonic Enhancers. (**A**) Shown is a flowchart of Venn diagrams showing the identification of 3,437 conserved blocks containing Su(H) sites in *D. grimshawi* and *D. virilis* with regulatory blastn (“*rblastn*”) E-value of <1e-15 using parameters calibrated to the NEEs. Homologous sequences from *D. grimshawi* were separated into test and control data sets for discriminative motif elicitation by maximum expectation (MEME) [33]. The test data set of 271 sequences contains Zelda sites (5′-CAGGTAR). The negative control set of 1,670 sequences is depleted of blocks containing any Zelda site (5′-CAGGTA, 5′-CAGGCAR, and 5′-TAGGTAR) or more than a single Su(H) binding sequence (5′-GTGnGAA). (**B**) The distribution of E-values for *rblastn* hits of the Su(H) strings shows that the test data set of conserved blocks containing Zelda sites are not any less conserved than the control data set lacking these sites. (**C**) MEME analysis identifies CA-dinucleotide and CAR-trinucleotide MSR motifs as being enriched in the Zelda+ dataset relative to other Su(H)-containing conserved blocks.

We then took these ∼3400 sequences from *D. grimshawi* and split them into two distinct sets (Fig. 6A). The first set of 270 sequences were identified because they had perfect binding sites for the embryonic temporal activator Zelda (5′-CAGGTAR), a pioneer TF for early gene activation [29]–[31]. As seen in the sequence alignments between the two Hawaiians and other *Drosophila* genomes, Zelda binding sites (Fig. 5 E, F, cyan nucleotides) are readily apparent between diverse MSR signatures (yellow green sequences in Fig. 5). The second set of 1671 sequences was derived by depleting the set of ∼3400 conserved blocks of those blocks containing either Zelda binding sequences (5′-CAGGTA, 5′-CAGGCAR, or 5′-TAGGTAR), or more than a single Su(H) binding site (5′-nGTGnGAAn). To ensure that our test and control data sets contained sequences with equivalent levels of conservation, we plotted the distribution of E-values and found that there are still proportionally many highly conserved sequences in both data sets (Fig. 6B).

We find that the Zelda-positive data set contains much more (CA)_n_- and (CAR)_n_- MSR content than the control data set using a discriminative MEME [33] analysis (Fig. 6C). (CA)_n_-MSR is known to be highly enriched in NEEs, and even enriched in *D. virilis* relative to *D. melanogaster*, which has a smaller genome [5]. As CAG-trinucleotide repeats and repeat instabilities are often seen in the protein-coding sequences for many transcriptional activators [34]–[37], we asked whether the enrichment of this motif was possibly due to the occurrence of nearby protein-coding exons near intronic enhancers. We find that almost all of the (CAR)_n_-MSR content contributing to the enrichment occurs in non-protein-coding sequence (see File S3 and Table S1).

In sum, these findings suggest first that Notch-target enhancers that are operative in the early embryo are more divergent (and hence potentially faster-evolving) than non-embryonic enhancers containing Su(H)-binding sequences, and second that the divergence is driven in part by a molecular drive mechanism that changes the internal spacing separating TF binding sites. In the Discussion, we propose some molecular phenotypes that could explain why natural selection would act on the prodigious output of this MSR drive mechanism.

### MSR-Enrichment in A/P Embryonic Enhancers of Hawaiian *vs.* Non-Hawaiian *Drosophila*

**Figure 7:**
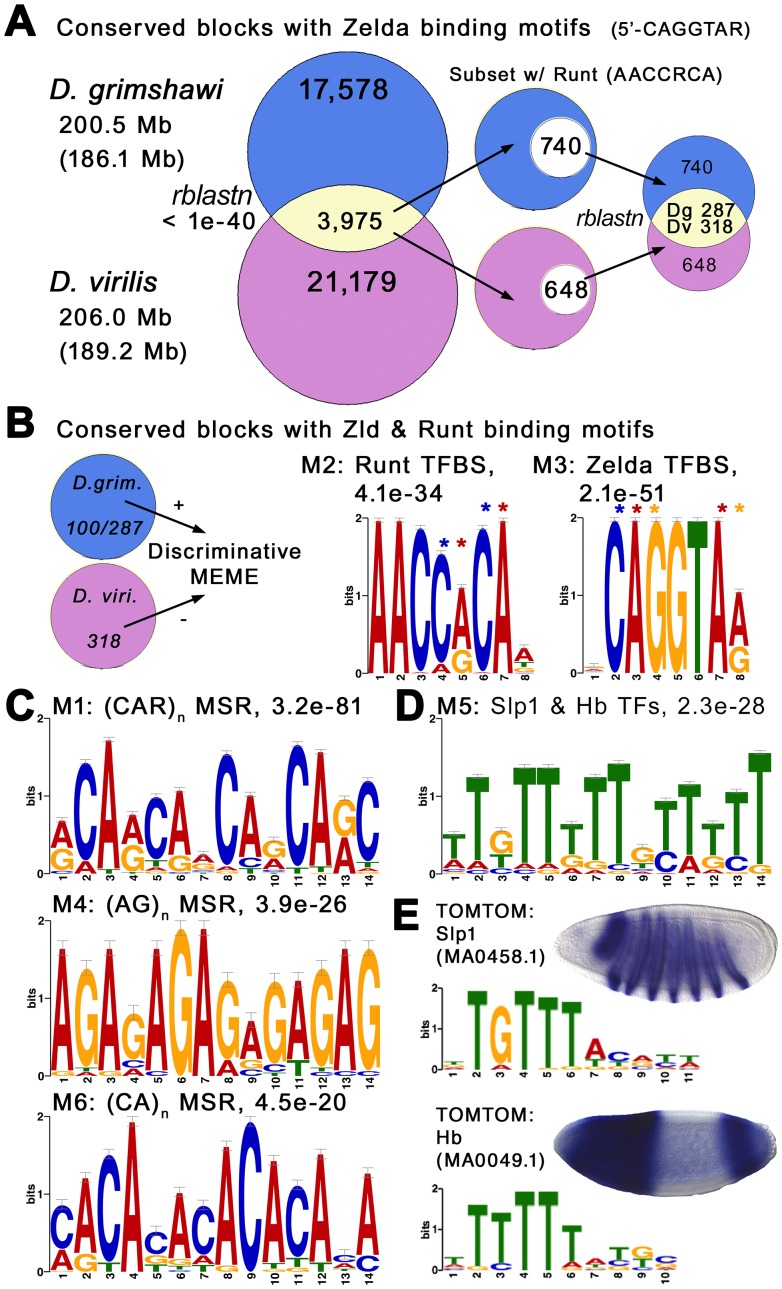
Diverse MSR Motifs are Enriched in the Embryonic A/P Patterning Enhancers of *D. grimshawi* Relative to their Homologous Sequences in *D. virilis*. (**A**) Shown is a flowchart of Venn diagrams showing the identification of 3,975 conserved blocks containing Zelda binding sites in *D. grimshawi* and *D. virilis* with an *rblastn* E-value of <1e-40. From these were chosen those that also contain a binding site for Runt (5′-AACCRCA), which is known to repress the posterior expression domains of targets induced by intermediate levels of the Bcd morphogen gradient. Because the Runt binding sites are not always found in the homologous sequences (either for lack of conservation or due to flanking truncation of the blocks), a second *rblastn* is performed to identify only the ones with reciprocal homologs. (**B**) A discriminative MEME analysis identifies the binding sites for Runt and Zelda relative in *D. grimshawi vs. D. virilis*, likely due to increased homotypic site clustering of these sites as well as (**C**) diverse MSR motifs, and (**D**) a motif matching binding sites for the pair-rule and gap products Slp1 and Hb. Numbers within each circle in (**B**) represent number of sequences used in MEME analysis due to constraints on test set (100 random sequences for *D. grimshawi*) *versus* control data set (entire data set or 318 sequences for *D. virilis*). Asterisks in (**B**) indicate di- and tri-nucleotide patterns found in the MSRs. (**E**) TOMTOM results showing matches to motif M5, and expression of *slp1* and *hb* in early embryo.

To determine whether unique MSR signatures are also enriched in embryonic, anterior/posterior (A/P) patterned enhancers, we first identified 3975 conserved blocks containing Zelda binding sites in *D. grimshawi* and *D. virilis*, with *rblastn* E-values of less than 1.0e-40 (Fig. 7A). From these we chose the subset of sequences that also contain a binding site for Runt (5′-AACCRCA), which represses the posterior expression domains of Bcd targets in the intermediate regions of the Bcd morphogen gradient [38]. Unlike the Bcd binding motif, the Runt binding motif is better suited to our question because it is: (***i***) well-defined [38], (***ii***) less variable, (***iii***) not related to binding sites for a large family of TFs such as the homeodomain-containing TFs, and (***iv***) associated with enhancers reading the rate-limiting parts of the Bcd morphogen gradient [38]. Because Runt binding sites were not always found in homologous sequences (either for lack of conservation or due to location of a truncated block near the edge of a contig), we performed a second *rblastn* query to identify only those with reciprocal homologs (Fig. 5A). We then performed a discriminative MEME analysis [33] using the set of homologous Zelda+Runt-containing sequences from *D. grimshawi* and *D. virilis* as the positive and negative data sets, respectively.

We find that the binding sites for Runt and Zelda are enriched in *D. grimshawi* relative to *D. virilis* (Fig. 7B), and suspect this is likely due to increased homotypic site clustering of these sites [39]. This is consistent with higher rates of binding site turnover, and we hope to investigate this matter in later studies. We also identified diverse MSR motifs, including the (CA)_n_-dinucleotide and (CAR)_n_-trinucleotide motifs previously seen in the embryonic Su(H) data set (Fig. 7C). We note that the binding motifs for both Zelda and Runt contain fragments of these sequences (asterisks in Fig. 7B). We also identify a clear (AG)_n_-dinucleotide MSR motif (Fig. 7C), which best matches the binding site for *Trithorax-like* (*Trl*), which encodes the GAGA-binding factor GAGA that is expressed ubiquitously in the early embryo [40]. Thus, Zelda, Runt, and GAGA may be natural sources of functionally variant spacer alleles, much like the Twist-binding site of the NEEs [19]. This further supports our previously proposed hypothesis [5] that MSR-enrichment of enhanceosome-building enhancers is related to the intrinsic MSR-seeding capabilities of Activator TF binding sites. Last, we identify a T-rich motif, which is likely to also serve as a source of mono-nucleotide runs (Fig. 7D). We find that this motif best matches binding sites for the pair-rule and gap products Slp1 and Hb, consistent with their expression patterns (Fig. 7E). This is also consistent with Zelda’s role in early embryonic timing of gene activation, and Runt’s role in repressing the posterior borders of expression of Bcd targets in the central region of the embryo.

## Discussion

### A Model for How Certain Classes of Enhanceosome Scaffold Result in MSR-Enrichment

Previous analyses of *Drosophila* genomic sequences have demonstrated a non-random distribution of microsatellite repeat (MSR) sequences in *Drosophila* genomes [41]–[43], the presence of compound (*i.e.*, clustered) MSR tracts [44], and an unpredicted excess of long MSR sequences [45]. It was also previously shown that the length of a spacer DNA separating linked Dorsal and Twist activator binding sites sites in the Neurogenic Ectoderm Enhancers (NEEs) can play a functional role [4]. It was then subsequently shown that CA-dinucleotide MSR related to the Twist site is used to source functional variants during evolution [5]. Here, we show that the Hawaiian NEEs offer an extreme case of MSR enrichment in terms of both the amounts and types of MSR content. These observations hint at additional spacer functionalities at other sites within the NEEs, the extent of functionality of which will have to be tested with additional mutagenesis in transgenic reporter assays.

Our results show that MSR-enrichment patterns can be linked to entire classes of regulatory DNAs, which in this case correspond to embryonic enhancer DNAs driven by the A/P and D/V morphogens patterning the syncytial embryo. Specifically, we showed that intervening DNA sequences between the Hawaiian *Drosophila* NEE binding sites have been replaced by microsatellite repeat (MSR) sequences and that these MSR sequences are still diverging. We showed that MSR demarcates the majority of the spaces separating the functional binding sites for Dorsal (*i.e.*, the *Dβ* site), Twist/Snail [*i.e.*, the *E(CA)T* site], Su(H), as well as the dedicated [non-*E(CA)T*] Snail binding sites, and sites for the general embryonic timing factor Zelda. Our use of footprinting to describe this effect of MSR enrichment in Hawaiian embryonic enhancers is in line with both phylogenetic footprinting and enzymatic footprinting. In MSR footprinting, phylogenetic footprinting, and enzymatic footprinting, the principle means used to reveal TF binding sites are by highlighting the space separating the sites *via* MSR content, divergence, and digestibility, respectively.

We also showed that the subset of conserved Su(H) site-containing Hawaiian blocks that contain binding sites for Zelda are specifically enriched in MSR motifs relative to non-Zelda containing conserved blocks from the same species, and that there was similar enrichment of MSR motifs in this set of Runt+Zelda containing blocks in the Hawaiian *D. grimshawi* relative to their homologous sequences in *D. virilis*. These results demonstrate that embryonic enhancers from Hawaiian *Drosophila* are enriched in MSR relative to both (***i***) other equally-conserved developmental enhancers from Hawaiian *Drosophila*, and (***ii***) homologous embryonic enhancers from non-Hawaiian *Drosophila*.

We propose that: (***i***) transcription factor activator complexes are sensitive to the spacing of Activator TF binding sites within enhancers that serve as assembly scaffolds for the aggregation of such complexes; and (***ii***) MSR enrichment in embryonic enhancers is a signature of frequent past selection for as yet unknown complex characteristics (*e.g.*, rate of assembly, complex stability on DNA, complex off-rate, and non-DNA bound complex half-life after assembly). As most indels are produced by microsatellite repeat slippage, enhancers from Hawaiian *Drosophila* lineages that are subjected to frequent evolutionary pressures would become grossly enriched in MSR content. Furthermore, as the embryonic enhancers would be subject to tremendously dynamic evolutionary pressures associated with both life cycle and ecological contexts affecting egg size, egg shape, and embryonic development during adaptive radiations, they would be more enriched in MSR signatures than enhancers operating at later developmental stages. While *Drosophila* lineages in general have exhibited much divergence related to adult pigmentation (*e.g.*, wing spot patterning), adult behavior (*e.g.*, mate choice and courtship song), and potentially other adult systems [46]–[52], we are unable to identify bioinformatically the enhancer sequences underlying these specific systems selectively without also pulling out many other non-changing adult enhancers. Thus, it is possible that the MSR enrichment we have seen for embryonic enhancers relative to all non-embryonic enhancers could be comparable to a select subset of enhancers underlying dynamically evolving adult phenotypes. Recent studies have indicated the utility of such MSR signatures for enhancer class identification [5], [53].

The genetics of microsatellite repeat number has received much attention also because of the role played by CAR-trinucleotide expansions in many neurodegenerative disorders and this has been extended to *Drosophila* [54]. In a study of length variation and evolution of CAR-trinucleotide microsatellite, or rather their “extreme conservation” in the *Drosophila* gene *mastermind* (*mam*) gene it was suggested that there must be strong selective constraints acting on the spacer lengths [36]. In a test of the null hypothesis that such length divergence arose by chance led to the conclusion that the CAR-MSR content in *mam* evolves both by molecular drive due to frequent repeat slippage and by natural selection on optimal spacer lengths [37]. Sequence data on *de novo* mutations from the HapMap project has also established that MSR-instability is repeat length dependent because similar instabilities are seen across diverse repeat unit sizes and sequences [34]. As we have previously shown the importance of CA-microsatellite repeat slippage emanating from the CA-dinucleotide rich Twist binding site in the NEEs [5], we conclude that MSR repeat variants are generally sourced by selection to adjust functional spacers across both cis-regulatory and protein-coding components of a genome. Thus, routine methods in bioinformatics, such as genomic repeat filtering and genome assembly based on point differences relative to a reference genome (as opposed to *de novo* assembly), may filter out important MSR-based functional variation that differentiates closely related genomes.

## Methods

### Bioinformatics

UNIX-shell scripts were written using grep, perl, and the BASH command set to identify all Su(H) sites and site clusters in the genome assemblies of *D. grimshawi* (r1.3) and *D. virilis* (r1.2) (see File S1). Site clusters were defined as two or more Su(H) sites located less than twice the desired flanking distance. For Su(H) binding sites (5′-YGTGRGAA) this was defined as 292 bp because (292 bp×2 flanking sequences) +8 bp = 600 bp. For Zelda (5′-CAGGTAR) we defined blocks as +/−300 bp from the Zelda binding site. The special case of not having enough flanking sequence due to proximity to the edge of a contig was also handled and these sequences kept in the data sets. For *blastn* analyses, the UNIX command line version of blast tools was downloaded from NCBI. The parameter set used for *Drosophila* enhancer bioinformatics identified largely by trial and error is the following: “-penalty −4 -reward 5 -word_size 9 -gapopen 8 -gapextend 6 -xdrop_gap_final 90 -best_hit_overhang 0.25 -best_hit_score_edge 0.1”. The subset of conserved Su(H) blocks with linked Twist–Dorsal sites (5′-CACATGT 0–41 bp GGAAABYCC) were identified with the UNIX-style regular expression: “CACATGT.{0,41}GGAAA[∧A][CT]CC”. All shell scripts are provided in File S1.

### MEME analyses

We performed discriminative motif discovery using Multiple EM for motif elicitation (http://meme.nbcr.net/meme/) and a control data set of negative sequences, and searched for “zero or one” occurrences per sequence [33]. We specified motif limits of 6 to 14 bp, and asked for an optimum number of sites between 10 and 300 with the upper limit varying depending on the size of the test data set, usually setting it at a maximum of 1.5x the number of sequences. For control data set we chose to use the maximum allowed dataset size of 240,000 characters. For the test data set limit of 60,000 characters we would choose a random sample if the data set was larger. For example, in the analysis depicted in Figure 7, we used 100 random sequences out of the 287 sequences available due to constraints on test data set.

### In situ hybridization

Whole-mount anti-sense in situ hybridizations with a digoxigenin UTP-labeled anti-sense RNA probe against *lacZ* were conducted on fixed embryos collected over a four hour egg-laying period held at room temperature. NEE reporters were integrated into the P-element vector between the mini-*white* gene and −42 *eve:lacZ* reporter as previously described [55].

### Molecular cloning

Live *D. mimica* were obtained from the UCSD stock center and reared with a protocol similar to that supplied from the stock center. Genomic DNA for PCR amplification was prepared using the Ashburner protocol [56], except that three adult flies instead of a single one were homogenized in a 1.5 mL microcentrifuge tube. Homogenization buffer, lysis buffer, and 8 M K acetate were used as described, followed by phenol-CHCl_3_ extractions, and EtOH precipitation. A 626 bp fragment of the *rho* NEE from *D. mimica* was cloned using the following oligonucleotide primers based on the *D. grimshawi* reference genome: 5′-AGA TGA AAA TCC GCA ATG CAA CGG (top strand primer), and 5′-AAA CAC AGC AGA AAG TCT CAA GC (bottom strand primer). A 513 bp fragment of the *vn* NEE from *D. mimica* was cloned and sequenced using the following oligonucleotide primers based on the *D. grimshawi* reference genome: 5′-ACA GAA GCT CAG CAT TTG GC (top strand primer), and 5′- GCC AGC GGC AAT TTT ATC TGC (bottom strand primer). A ∼500 bp fragment of the *vnd* NEE from *D. mimica* was cloned and sequenced using the following oligonucleotide primers based on the *D. grimshawi* reference genome: 5′-CCA CCG GGT CTC AAA TTC TTT CAC AGT (top strand primer), and 5′-CCA CCG GGT CTC AAA TTC CCA TCA ACA (bottom strand primer). These amplified PCR fragments were cloned into Promega’s pGEM-T easy cloning vector. Clones were sequenced, and a few were selected to be cut with EcoR I, gel purified, and ligated into the EcoR I-cut pCaspeR P-element vector carrying the −42 *eve lacZ*-*tubulin* 3′UTR reporter construct previously reported [4], [5]. The cloned enhancers from *D. mimica* have been deposited at GenBank and have accession numbers: KJ814003 (Dmim_rhomboid_NEE), KJ814004 (Dmim_vein_NEE), and KJ814005 (Dmim_vnd_NEE). In addition, the sequences for the NEEs of both Hawaiian *Drosophila* species are included in File S2.

## Supporting Information

Table S1 Location of CAR repeat-rich sequences in conserved Su(H) blocks.
(PDF)

File S1 Unix computer scripts.
(TGZ)

File S2 FASTA file of *D. mimica* and *D. grimshawi* enhancer sequences.
(TXT)

File S3 Annotated Su(H) blocks from D. grimshawi (Zelda+) with (CAR)_n_ repeats.
(PDF)
