# Supplementary material for: Microsatellite Repeat Instability Fuels Evolution of Embryonic Enhancers in Hawaiian Drosophila
Source: PLoS One. 2014 Jun 30;9(6):e101177. doi: 10.1371/journal.pone.0101177 (PMC4076327; doi:10.1371/journal.pone.0101177)
Supplement: Table S1 — Location of CAR repeat-rich sequences in conserved Su(H) blocks. (PDF) [file pone.0101177.s001.pdf]

Microsatellite repeat instability fuels evolution of embryonic enhancers in Hawaiian *Drosophila*

Andrew Brittain, Elizabeth Stroebele, and Albert Erives

Supporting Information:

Table S1. Location of CAR repeat-rich sequences in conserved Su(H) blocks.

**lcl**	**Location of lcl_# sequences with CAR-MSR in genome annotations**	**Repeat length (# of CARs)**	**$(CAR)_n$ Repeat Location**
1025	Intergenic, intronic to *D. mela. Snoo*	8 and 10	Intronic
1726	Intergenic	5 and 6	UTR
2862	Intronic to hypothetical gene	7	Intronic
4636	BOTH intronic/exonic, in *D. mela. wb*	6	Intronic
6209	Intronic, in *D. mela. mamo*	6	Intronic
9220	BOTH intronic/exonic, in *pnt*	6	Exon
9335	BOTH intronic/exonic	6	Intronic
1759	Intergenic, near *Klu*	5	UTR
10728	Intronic, in *Ptx1*	4	Intronic
4177	Intergenic, intronic to *D.mela. Shroom*	4	Intronic
8168	Both intronic/exonic, in *vnd*	4	Intronic
8914	Intronic	4	Intronic

Sequences with > 4 CAR repeats
In: "35_dgrim_e-30_99_SuHZld.txt"
