# Supplementary material for: Microsatellite Repeat Instability Fuels Evolution of Embryonic Enhancers in Hawaiian Drosophila
Source: PLoS One. 2014 Jun 30;9(6):e101177. doi: 10.1371/journal.pone.0101177 (PMC4076327; doi:10.1371/journal.pone.0101177)
Supplement: File S3 — Annotated Su(H) blocks from D. grimshawi (Zelda+) with (CAR)n repeats. (PDF) [file pone.0101177.s004.pdf]

Microsatellite repeat instability fuels evolution of embryonic enhancers in Hawaiian *Drosophila*
Andrew Brittain, Elizabeth Stroebele, and Albert Erives

Supporting Information:

File S3. Annotated Su(H) blocks from *D. grimshawi* (Zelda+) with $(CAR)_n$ repeats.

Cyan and yellow sequences indicate location of tracts on top and bottom strands, respectively.

```
>lcl_1025 SuH_block_type_SFf downstream
TTTCTCAAGAGAATTTTGCACACGTGCAAGTTGCAGCAGAGGCTGAGAGGCTGCTACCTGCGTAGGAAAACCCTTAA
TGGGCAGCAACAACAACAACAACAACAACAACAAGAAGAAGCAGAAGGAGAAAGAGAGCGAAGAGAGGCAGACAATG
GAAATGCGTGAGAAAACTCGTTATGACCTGCACGGGAAGGCATTATAATTCCCCTAGTTGTTGTTGCTGTTGTTGTT
GTTGTTTGCCGCAATTCGTTGCACTTGTTTACATGGGAAATGATTTTTCACATTGTTAACGCTCAGTTTTCCTCATA
ATTTTCCATCAGCTACTGCT
--
>lcl_10728 SuH_block_type_SSFf first intron (Ptx1)
AGATCGATGACGTTAGTTTTCCCAACGATGATGATGATGACGATGATGATGGTGCTGAGCAATGTACCTTGACAAAT
TGACACATTGCTGCTGTTGGGCAATATTGAAATTGGCCAAGACTTTGATTTATGCCCTGTTTGCAGCAACTTAAAGG
TTGCCCTTCTCACGCTGACAATATTTAACCAGAGTCACAGCTCAATCTCTGTGTGGCTCATGCGATTTCAATTAACT
GAGCGCAACTAATTCGATTAAGGCAATTACCAAGCAAATAAATGCGCATTAAATTGGCCACGAGCCAAACAACAACA
ATCCAAAAACTTTGACTCACTTGTCAAAGTTCAACTTCGGTTCCCACAAAACAAACAAAACATTTTCAACCAGACAG
TTCAGTTTTAGGCCCAGTTTTTGCATACATACGCGTGTCCACATTTAGTTACGGCAGGCAATGTTGCACGAGGCACG
AGACAAGGCCCATAAATTGCAACCCCAGTGAATTGTGCGCTGATTTAAGATACA
--
>lcl_1726 SuH_block_type_SSFf upstream
TTTTACCTGATTTCGACGCGACGGCACACAAACTTACCAAACATATTCGCCCTCAATGTCACAACAACAACAGCAAC
AACGCACAGCACATGGCTCGGGCTCTGTTTTGTTGTTGTTGTTGTAACATTCGTAGAAATCACAAGTGCCAAGCGCG
CTTCATTGTGGGAACTGTTTTTGGAGTTGGATCGCTTCTGGTGCAAGTATCTGCCCCTCAAACCCTCCCTCACACAC
ACTTCTTCTCTCCCTCCCTCTCACTCTCTCTCTCTCTCTCTCTCTCTCTATTTATATATTTAGTGTTTTGGGCAC
TTTGGGCCTCTTCACATATTGACTCTAATATCCTTTTGAATTTGGTTTGATTAACCTTTTGATGATCAATGATAATG
AATATTTATTAACTGTTCGCCGAGCGCCATAAATTCCCACACTTTGTTGTGTCCAAACATGACATATTAAAAAAACA
CTTTAAACATTAATTTTGAAATACAAATATAACGATGGATATATTTTAAGTAGCTCTGTTGTGAAACTATTTCCTGT
TGTAACAGTTAACATTTGATAACTGCCATTAAAAACGAGAAAACTTA
--
>lcl_1759 SuH_block_type_SFf upstream (Reg-2)
ATCAACTTTTTTATGCATTTCTTGCCTTTCCGAGTAAAGAAAAAAACAATTTGGCAACTTGGGGCAGGTAGACAAAG
AGATTTGTTTGGTAGGTCAAAACGACAGAAACGTGCAAGTGCTGTTATTGTTGTTGTTGTTGTGTTCGTCTGATTGT
TATTGCTTCCCACGAGCATTAATAAAACTGACACGTTTTCGCATATGCCAAGTTGCTTCTCCACATTTATTTTTGGC
AAAAAAAAACGTTGCCGACCTCGAAATGGCAACGGCGCCAAATCGAACGTTGCACATAACAAACAATCAACTCTTCC
CGCTTCAAATCCATTTTCAA
--
>lcl_2862 SuH_block_type_SFf intron
TTCCAACATTTTTTTGCTACTTGTTTTGAGCCCAATCACGTTGTCTACAAGACATTTAACCCATTTGTAAAAGGCAG
TCTCCACTCCCCATAAAAATATGCAAATTACCTGGCGCATCTGGACAGGCGACTGTGGCAGCTTCAACATTAACTGC
AACCACTTCTCACACCAGACACCGCAACAGCAAAGCCAACAACAACAACAACAACAATCGATAGCTGGTGGCAATAA
ACGCAGCAATTACACAGTTGGCTATTCTAATTCACTCATTTTTGGCAAAAATAGGTGCGCCCCGCATCGATGTCTGG
GGTTTTGCCATTGGGGTTTC
--
>lcl_4177 SuH_block_type_SFf upstream
TGAACGCTTATGTTGTGATTAGGTTTACCTGTGAGCTCTGGACTCAGTTTGCATTTAGCTGCAAAGGCTGCCTTAAG
CGCTGCTAGCTGCTAGCTGCTAGCTGTTTGCTGTTTGCTGTTAGCTCTTTGCTGCTGCTGCGATTCATTATAATTTT
TGTCAATTCTCACAGGTAGCGCGCTGAGCGGACAAAGGTGAAGATCGCATGGCGCTGAGACATTCACACAGCGCTAA
CAGTAACCCCAACAACAACACGCATAAATTTACAATTAAATTTAATTCCTTCACTGGATTTCCGCAAAAAAAAGGCA
GCAAAAAAAGGGTTCCAACA
--
>lcl_4636 SuH_block_type_SFf Intron and exon (longest repeat NOT in exon)
TTTGGCACTAAACTGTCAGATCAGAATGGTCACAGCGAGATCTCAGGCACTTTGCTCTCGATGCTGTCTGGTTACAC
AACAGATGAATGTTGGCCAGGTAAAAAAAAAAAGCGTTCCAAAGTTGAGCGAAGCAACGGGCAAATGTCCAATTTAA
```

TTGTAATGTGAGAAATTTAAATTAGGTAGCCGCAAAATGCGGCTCAGATGGTTGAACAATGACTACGAAATCTCAAG
CGGAAGATGCCCGAAGAATACAACAACACACAAAAATTATGAAAAAAGAGTAAAAATGTTTCAACAACAATATATGT
GTTGTTGTTGTTGTTGTTGT
--
>lcl_6209 SuH_block_type_SFf intron
TAAAGTATTACAGAACCACGGGGACCGCTGAGGTCGCCCACGCGCCATTTGTGGCGAACGTGAACTTTGCGCGCCAA
CTGTATCTAGAAAATAGTATATATGTGTGATATATATTTGTACATAGATATACACACACACACATACATCTTTCCAT
ATCAGTTTCTCACGCTCACAATATGGTTTTTACAACAGGTAAAATGCCAGCCAAGATTTTGCCGTTTTTGGGCGCC
AGAAGGTTTTCTGTTGCCTGCGATTTTTGCGCTTACATGACAAGCGCCCGTTTGGGCCTTTTCATTTGTTGTTGTTT
CTGTTGTTGCTGTTGTTGTT
--
>lcl_8168 SuH_block_type_SFf intron and exon (vnd) (longest repeat in exon)
GACAGCTGACCACTACCTGGTTGGGATGCTCGTCTTCGTTTTGGCCAGGCTAGCACTAGCAGTAAACGTTAAGCATT
TTTCAGCCACATGTTGGCGCAAGTAAAAAGCGTTCTTGCTTCCGGTTTTTCCATGCTGAATTTTCCCAAATGTGCGC
GCGAAATTCCCACGCGCTCAGGTCCACATCAGGACGCGGGGCATTCCCCGCATATCTGAGCGGAATTCCCTTTGCAC
TCGTCCAACCTTCGGCGCAGACAGTGTGTCGCCGTTTTGCGTCTTGTGTTGTTGTTGCTGGTGCTGGTTCTGTCGCG
TCTCGTGTCTGCTGCACACA
--
>lcl_8914 SuH_block_type_SFf intron
TATTGCTGTTGTTGTGATGGGTGTGTCTGTGTATGTATGTGTGTATTTGTGTGTGTGTGTGTGTGCTTGTGTGTG
TGAGAGAAATATGCAGTCTACTCAATGGAGGTCGTTCGCTATTCTCCCTGCTTAGCATAAGGCAATTCCATTGTCTT
GTATCATGTGAGAAAACGTGTGCATTCTTAAGGTACCTTCAAATTTTTGTAATTATAAACATTAAGTGAGTCTGTGT
ATGTGTGTGAGTGTGTGTGTGTGCATTGGGGCCTATTAGAACTAGGTAACCATGAGTAAGATTACTTCTATGAAAAC
ATACAATTTTGTGCATTAGG
--
>lcl_9220 SuH_block_type_SSSFf intron and exon (pnt) (one repeat in exon)
CGCAAAAGAAGTTGAGTAAAAAATTAAATGAAATGAAATCAGTGCTTAAAGATTGAGGGGAGACAAGAGCCCCAGCA
GCAGCAGCAGCAGCTTGAGAATTTAGCCCAAAAATACCAAAAAAAAAAAGGCACACAAAAAGGGAAACCAAGAAATA
TTCGTATTCCCACGACTCATGGAGCCAGACGCCAACGCGACGAGTCCTTGATCGAGGCATTGAAATGTACGAGTACC
TCGGTAAGTGATAGTTGCTCTCAGATTAAATCGTCTGCATTCGCAATGATTCGCACATTGGCCAGCAAGTCGAGCAT
ACCGCAAACCGGCATGCAGGGTATTCACCTCTTCGACAATTCACTCAGTCTCTTAAAGAGCCCTTTTCTTTTTGGGG
GGATTAGCAGGTAAAAGCCGAAAGGCAGCCTCATTGTGTACCGCTCACAGGATCCGCAGAGACGACGAGTGTGTCTT
TGTAATTCGAGTCGAACCATTCATTTTTTCGGGTTTTTATTTCATCTTTGCCTAAAACAAATATGAGCATATATATT
AAAACACAGATGTATGTAAAATGCTCGCCAAAAAGAATTTCAAGTTCAAGTGCGTGGGAATTTTGAGTTCTGCGAAT
TTTCATGGTCTGTAGAATATCATAATGGGGATTGATGGTCAATTGTACGTCTATTAATGTATAATATTAAATTGAAT
TTAAATTTCTTGATACCTTCACAACATTTTCTTATTGAATTAAATATAAAAATAAATCCCTTGAGCATAATTAATTA
GTAATGTGTATATAAAAGGACAATTATCACACGGCTCAGGTGCAGCATTTTTCGTTTCTCTGTGGCTGCTACCCTCA
TCAAAATGGCAATTAATTATATCATTTTTTTGAATGATTGTCTTCTACTCCAAATATTCGAAACGTTCGTATTGGGC
TCGATCGTTTTGCACCCTGTTTGCGGGGCACGCACGGGCGATCCTCTCTGAATCTTGCAGCATGCAACATGTGAGAA
ACAAACACACAATCGCGCACACACACACATATATGCACACTCACACACACACACACACACAGGTTCGCCAAGACGGG
CATACACAATTTAAGTGTTGAGTGTGTAATTTTATATTTCCGGTGTAGTAAGTTTCATACGACAACTCAGCTCTTTG
CCTGGG
--
>lcl_9335 SuH_block_type_SFf intron and exon (CG1499_RA or CG1499-RB) (repeat NOT in exon)
ATTTATGCGCCTGGGTCTTGTGGCCACCATGAACTAGAACTCAGTAATTGAATGAATGAGCCGACCAGGTAAAATTA
TTGAACTTTCTTTGGCACACAAAACAGGCAGCTGCATAAAACGACAGCAGCATCAGCAGCATCAGCAGCAGCAACTG
TTGAAATTCTCACACTTTCTAACTCACCTGGCAATTTATCGGCGGTCGTCTTGAACAGCACACACAAGCCGGTTTCA
TAGTTGACAGAGCTACAGGATTCGTTGCTCTGACAGGCCTCCAAGCAGTCCGTAAGCATCAGAGTACCCGGCAATGT
ATCCAACATTTTTGAGGGCG
